# Dual-modified liposome codelivery of doxorubicin and vincristine improve targeting and therapeutic efficacy of glioma

**DOI:** 10.1080/10717544.2017.1344334

**Published:** 2017-07-07

**Authors:** Yue Zhang, Meifang Zhai, Zhijiang Chen, Xiaoyang Han, Fanglin Yu, Zhiping Li, Xiangyang Xie, Cuiyan Han, Lian Yu, Yang Yang, Xingguo Mei

**Affiliations:** a State key Laboratory of Toxicology and Medical Countermeasure, Department of Pharmaceutics, Beijing Institute of Pharmacology and Toxicology, Beijing, PR China;; b Hubei University of Science and Technology, Xianning, PR China;; c Jiamusi University, Jiamusi, PR China;; d Outpatient Department of Beijing Space City, Aerospace Systems Divison, PLA Strategic Support Force, Beijing, PR China;; e Department of Pharmacy, Wuhan General Hospital of the Chinese People’s Liberation Army, Wuhan, PR China;; f School of Pharmacy, Qiqihar Medical University, Qiqihar, PR China

**Keywords:** T7 peptide, ^D^A7R peptide, brain targeted drug delivery, glioma, doxorubicin, vincristine

## Abstract

Therapeutic outcome for the treatment of glioma was often limited due to drug resistance and low permeability of drug across the multiple physiological barriers, including the blood-brain barrier (BBB), and the blood-tumor barrier (BTB). In order to overcome these hurdles, we designed T7 and ^D^A7R dual peptides-modified liposomes (abbreviated as T7/^D^A7R-LS) to efficiently co-delivery doxorubicin (DOX) and vincristine (VCR) to glioma in this study. T7 is a seven-peptide ligand of transferrin receptors (TfR) capable of circumventing the BBB and then targeting glioma. ^D^A7R is a d-peptide ligand of vascular endothelial growth factor receptor 2 (VEGFR 2) overexpressed on angiogenesis, presenting excellent glioma-homing property. By combining the dual-targeting delivery effect, the dual-modified liposomes displayed higher glioma localization than that of single ligand-modified liposomes or free drug. After loading with DOX and VCR, T7/^D^A7R-LS showed the most favorable antiglioma effect *in vivo*. In conclusion, this dual-targeting, co-delivery strategy provides a potential method for improving brain drug delivery and antiglioma treatment efficacy.

## Introduction

Glioma remains one of the most devastating malignant primary brain tumors in humans with a median survival after maximal therapy of less than 2 years after first diagnosis (Dolecek et al., 2012). The special pathological and physiological characteristics make glioma treatment very difficult. Due to the infiltrate growth of glioma, it is extremely difficult for surgical resection to completely eliminate glioma (Donahue et al., 2008). Whether the operation is available or not, chemotherapy is the most common method for the treatment of gliomas (Liu et al., 2012). However, chemotherapy is often hindered by the low permeability of drug across the multiple physiological barriers and the drug resistance (Shaw et al., 2017).

A major hurdle in the clinical management of glioma is the presence of the blood-brain barrier (BBB) which excludes more than 98% of small molecule drugs and almost 100% of large molecule drugs. In order to meet this challenge, many attempts have focused on the conventional nanocarriers (e.g. liposomes, polymeric nanoparticles, and polymeric micelles), which could make a significant contribution to increase the permeability and retention (EPR) effect and cross the physiological barriers. However, because of a lack of specific targeting, it is difficult for these nanocarriers to effectively accumulate in brain tumors merely based on their nano-size. Moreover, EPR effect depends on the degree of vascularity and endothelial fenestration (Lee et al., 2017), it often works in rodents but seldom in humans (Danhier, 2016). Thus, various targeted strategies have been applied in the field of nanocarriers to overcome physiological barriers and target the glioma (Qu et al., 2016). In recent years, actively targeted drug delivery systems have demonstrated the potential to improve the outcome of glioma therapy. A unique targeting agent, T7 (HAIYPRH), which was recently identified by phage display, could bind to transferrin receptors (TfR) with high affinities and specificities (Shinde & Devarajan, 2017). Since TfR are both overexpressed in brain endothelial cells and brain glioma cells, T7 peptide has been used as a ligand in targeted drug delivery systems for the BBB penetration and glioma therapy (Kang et al., 2015).

Another barrier is the blood-tumor barrier (BTB), which restricts accumulation of drug in tumor, exacerbating the failure of chemotherapy (Gao et al., 2014). The BTB of brain tumors is tighter than the BTB of peripheral tumors regarding the transendothelial fenestrations, transporter expression, and interendothelial cell gaps (Song et al., 2016). Vascular endothelial growth factor receptor 2 (VEGFR 2) is highly expressed in brain glioma cells and implicated in tumor angiogenesis, growth, and metastasis (Binetruy-Tournaire et al., 2000; Niu & Chen, 2010). Therefore, VEGFR 2 becomes an ideal target for glioma-targeted drug delivery. d-peptide ^D^A7R (^D^A^D^T^D^W^D^L^D^P^D^P^D^R) screened by a phage display system has a high affinity for VEGFR 2. More recently, it was reported to ^D^A7R-modified liposomes could efficiently inhibit glioma growth by crossing the BTB and targeting glioma cells (Ying et al., 2016).

Moreover, single-drug therapies for the treatment of cancers are ineffective over a long period of time (Morton et al., 2014). The mechanisms of drug resistance include some enzyme inactivation, membrane permeability changes, blockage of drug into the target structure, the original metabolic process alterations, and so on (Liang et al., 2010). Combination therapy seeks to increase cancer eradication efficacy without amplifying systemic toxicity while simultaneously overcoming drug resistance. Recently, various combinations of drugs have been developed for treating various cancers targeting various intracellular components (Wu et al., 2016). Doxorubicin (DOX) and vinca alkaloid vincristine (VCR) are commonly used antitumor drug. However, clinical utility is hampered by cardiotoxicity and drug resistance (Arnold et al., 2004). When be administrated with DOX, VCR exerts cardio protective effects on cardiac myocyte toxicity induced by DOX, as well as chemical and hypoxic oxidative stress (Takemura & Fujiwara, 2007). In this way, DOX and VCR act on different targets of tumor cells; their combination would not only effectively surmount the drug resistance, but reduce the cardiotoxicity as well. For the synergistic effect of DOX and VCR, their optimized dose ratio was measured to be 4:1 as many clinical trials report (Elias et al., 1978; Arndt et al., 1998; Augustinus et al., 2001; Hofmeister et al., 2008; Kawasaki et al., 2014).

Those functional nanocarriers could circumvent the one or two barriers to improve the antiglioma effect *in vivo*; however, an ideal glioma-targeted drug delivery aiming to overcome all of the aforementioned hurdles has not been achieved so far. Herein, we hypothesize that systemic glioma-targeted drug delivery would be achievable by using the dual-targeting (T7 and ^D^A7R peptides) and combination therapy (DOX and VCR) strategy to overcome the multiple physiological barriers (BBB and BTB) and drug resistance. T7 and ^D^A7R were modified on the surface of PEGylated liposomes (abbreviated as T7/^D^A7R-LS) containing DOX and VCR. In this study, the efficacy of T7/^D^A7R-LS in crossing the BBB/BTB and co-deliver DOX and VCR were assessed *in vitro* and *in vivo*. Moreover, the *in vivo* targeting efficiency, synergistic tumor suppression effect of the liposomes was evaluated on the C6 tumor mice model. The findings have provided valuable preclinical data to validate a noninvasive, efficient targeted peptide-nanotherapy for treatment of glioma, one of the most untreatable and deadly malignant diseases.

## Experimental materials

### Materials

T7 and ^D^A7R were synthesized by Cybertron medical technology Co. (Beijing, China). Hydrogenated soy phosphatidylcholine (HSPC) and cholesterol (Chol) were purchased from Lipoid GmbH (Mannheim, Germany). 1,2-distearoyl-sn-glycero-3-phosphoethanolamine-N-methoxy (polyethylene glycol) (ammonium salt) (DSPE-mPEG_2000_) and 1,2-distearoyl-sn-glycero-3-phosphoethanolamine-N-maleimide (polyethylene glycol) (DSPE-PEG_2000_-Mal) were purchased from Xi'an ruixi Biological Technology Co., Ltd (Xi'an, China), Inc. (Alabaster, AL). Doxorubicin hydrochloride (DOX) was supplied by Haizheng Co. (Zhejiang, China). Sulfate VCR was obtained from Baiyunshan Co. (Guangzhou, China). 4,6-Diamidino-2-phenylindole (DAPI) was purchased from Beyotime (Haimen, China). All chemicals were of reagent grade and were obtained from Sigma-Aldrich (St. Louis, MO), unless otherwise stated.

Human umbilical vein endothelial cells (HUVECs), glioma C6 cells, and mouse brain endothelial bEnd.3 cells were provided by the Cell Resource Centre of IBMS (Beijing, China) and cultured in Dulbecco’s modified Eagle’s medium (DMEM) containing 10% FBS (Gibco, Carlsbad, CA).

Female ICR mice (weighing 19–21 g) were purchased from Vital River Laboratories (Beijing, China). All animals were handled according to the code of ethics in research, training and testing of drugs as laid down by the Animal Care and Use Ethics Committee of Academy of Military Medical Sciences.

### Methods

#### Synthesis and characterization of targeting molecule conjugates

DSPE-PEG_2000_-T7 and DSPE-PEG_2000_-^D^A7R were synthesized by conjugating DSPE-PEG_2000_-Mal to the cysteine residue on T7 and ^D^A7R, respectively. Briefly, T7 and ^D^A7R were conjugated with DSPE-PEG_2000_-Mal (1:1 molar ratio) in chloroform that contained triethylamine (TEA, 5 eq.) at room temperature for 24 h while stirring. The reaction mixture was dialyzed (molecular weight cutoff (MWCO) 3.5 kDa) in distilled water for 48 h to remove the chloroform and unreacted peptides. The final solution was evaporated by rotary evaporation and stored at −20 °C until further use. The existence of the conjugations was confirmed with MALDI-TOF mass spectrometry (MALDI-TOF MS).

#### Preparation of liposomes

The non-modified liposome (N-LS) with composition of HSPC: Chol: DSPE-PEG_2000_ (mol/mol, 50:43:7), were prepared by direct hydration of a lipid film as described previously (Yang et al., 2015), with minor modifications. Briefly, all lipids or hydrophobic probe (Cy5.5) were dissolved with chloroform-methanol (3:1, v/v) in a pear-shaped flask and were subsequently evaporated to form dry film using a rotary evaporator under vacuum. The lipid film was then hydrated using 300 mM citric acid buffer solution at 50 °C for 30 min. To control for the size, the lipid dispersion was extruded 11 times through 100 nm polycarbonate filters using a mini extruder (Avanti, Canada). The T7-modified liposomes (T7-LS) and ^D^A7R-modified liposomes (^D^A7R-LS) were prepared followed the same procedures, except the DSPE-PEG_2000_ was partially substituted by DSPE-PEG_2000_-T7 and DSPE-PEG_2000_-^D^A7R conjugates (molar ratio, 0.5, 1, 2, 3, and 4%), respectively. For T7/^D^A7R-co-modified liposomes (T7/^D^A7R-LS), the content of both conjugates was 3%.

DOX and VCR (4:1, w/w) were loaded into various liposomal formulations using the pH gradient method at 1:20 Drug/Lipids mass ratio as described by our previously report (Li et al., 2016). Briefly, blank liposome suspension prepared as described above was directly added into 300 mM citric acid buffer solution, added into DOX and VCR to obtain the drug concentration of 2.0 and 0.5 mg/mL, respectively; then adjusted pH of extrinsic phase to 7.5 using 100 mM Na_2_CO_3_ solution, and incubated at 55 °C for about 30 min. Finally, the drug-loaded liposomes were filtration sterilized by 100 nm polycarbonate filter and subpackaged to aseptic pials (DOX 1.6 mg/mL, VCR 0.4 mg/mL).

#### Characterization of liposomes

The mean diameter and zeta-potential of each formulation was determined in three serial measurements using a Malvern Zetasizer Nano ZS90 instrument (Malvern Instruments Ltd., United Kingdom). The morphology of T7/^D^A7R-LS was observed via transmission electron microscopy (TEM, HITACHI, H-7650, Japan) and atomic force microscopy (AFM, NanoWizarc, JPK Ltd., Germany). The *in vitro* stability of liposomes were evaluated using a Turbiscan Lab^®^ Expert (Formulaction, L’Union, Toulouse, France), an innovative analytical instrument able to determine the small changes of colloidal systems. T7/^D^A7R-LS was diluted by cell culture medium (90% DMEM) containing 10% FBS and analysis for 72 h. Measurements were carried out using a pulsed near infrared LED at a wavelength of 880 nm. The VCR and DOX encapsulation efficiency (EE) of each formulation were determined by HPLC as described by previously report (Dong et al., 2016).

#### Effect of peptide density on cellular uptake of liposomes

To investigate the effect of T7 and ^D^A7R peptides density on cellular uptake, Cy5.5-loaded mon-modified liposomes T7-LS and ^D^A7R-LS) were prepared at different peptide densities (0.5, 1, 2, 3, and 4%, molar ratio). C6 cells were seeded at a concentration of 5 × 10^5^ cells/well in six-well plates for 24 h. Then, the cells were incubated with different liposomal formulations for 2 h at 37 °C and the cells were rinsed with cold PBS, trypsinized, and washed three times with cold PBS. The samples were then centrifuged and resuspended with PBS. Approximately, 10^5^ cells were applied immediately using a flow cytometry (FCM) (BD FACSCalibur, Millipore Corporation, Billerica, MA). The concentration of Cy5.5 was 150 ng/mL.

#### Binding of different liposomes to cells

In order to assess the binding affinity of different liposomes to cells, different 5 μM Cy5.5-loaded mon or dual-modified liposomes were incubated with three types cells (C6 cells, HUVECs and bEnd.3 cells) at 37 °C for 2 h, respectively. The cells were washed three times with cold PBS, then centrifuged and resuspended with PBS for qualitative analysis by confocal laser scanning microscopy (CLMS) (UltraVIEW Vox, PerkinElmer, Waltham, MA) and quantitative analysis by FCM.

#### *In vitro* cytotoxicity assay

Cytotoxicity of free DOX + free VCR and various liposomal formulations containing DOX and VCR against C6 cells were evaluated with MTT assay. The cells were seeded into a 96-well plate at a density of approximately 4000 cells/well. Then cells were treated with various formulations at a range of concentrations. After the cells were further incubated for 72 h, 20 μL of MTT solution (5 mg/mL in PBS) was added to each well. After 4 h incubation, the percentage of cell viability was determined on the basis of absorbance at 490 nm by a plate reader (Model 680, BIO-RAD, Hercules, CA).

#### Transport across the *in vitro* BBB and BTB model

To establish *in vitro* BBB model, a bEnd.3/C6 co-culture BBB model was established according to previous reports (Li et al., 2014). Briefly, bEnd.3 cells were seeded on the upper side at 1.0 × 10^5^ cells per insert (Corning, NY). C6 cells were seeded on the basolateral compartment of the insert at 2000 cells/compartment. To establish *in vitro* BTB model, C6 cells were plated onto the lower chamber, and HUVECs were seeded into the upper inserts of transwell with a density of 5:1 C6/HUVECs ratio (Khodarev et al., 2003). After incubation for 5 d, these models were used for experiments. To assess the antiproliferative effect of the various liposomal formulations containing DOX and VCR against C6 cells after penetrating the BBB model or BTB model, a sulforhodamine-B staining assay was applied. Free DOX + free VCR or various co-loaded liposomes were added to the apical compartment of the BBB model or BTB model. The final concentration of DOX and VCR was 1000 and 250 ng/mL, respectively. After 48 h, the percentage of surviving glioma C6 cells in the basolateral compartment was determined by the sulforhodamine-B staining assay (Li et al., 2014).

#### Establishment of glioma-bearing mice model

Glioma-bearing mice were established as previously described (Gong et al., 2011). Briefly, the mice were anesthetized with a peritoneal injection of a solution of 10% chloral hydrate at a dose of 5 mL/kg. The posterior cranial region was shaved and prepared in a sterile fashion. After fixed on stereotaxic instrument, a midline incision approximately 0.5 cm in length was made at the convergence of the head midline and intercanthal line. A skull hole was drilled at bregma using a steel drill bit at 1.8 mm to the right of sagittal suture. C6 glioma cell suspension (4 × 10^5^ cells in 4 mL PBS) was injected using a 22-gauge 10 mL Hamilton syringe at a depth of 3.0 mm into the brain. After surgery, the animals were allowed to recover under observation and then returned to their cage.

#### *In vivo* imaging

Cy5.5-loaded liposomes were prepared for glioma-targeting evaluation. Fourteen days after surgery, the mice were administered via tail vein injection with Cy5.5-loaded different liposomal formulations. Four hours after the injection, the *in vivo* imaging was performed with an IVIS^®^ Spectrum-CT. Bioluminescent and fluorescent signals were quantified using Living Image^®^ software (Caliper, Alameda, CA).

#### Glioma distribution

Fourteen days after tumor implantation, the Cy5.5-loaded different liposomal formulations were administered via tail vein injection to the mice. After 1 h, the mice were anesthetized, and the hearts were perfused with saline, followed by 4% paraformaldehyde. The brains were removed for consecutively preparing 5 μm thick frozen sections. Nuclei were stained with 1 μg/mL of DAPI for 5 min. The distribution of fluorescence was observed using CLSM.

#### *In vivo* antiglioma effect

The glioma-bearing mice were randomly divided into the following five groups (10 mice per group): T7/^D^A7R-LS group, T7-LS group, ^D^A7R-LS group, N-LS group, free DOX + free VCR group, and 0.9% saline group. Eight days after cell injections, each mouse received a dose of 1 mg/kg (DOX 0.8 mg/kg + VCR 0.2 mg/kg) four times every 2 d. At day-16, four mice from each group were anesthetized and brain cancer assessed by magnetic resonance imaging (MRI) (Siemens, Munich, Germany) by measurement of tumor diameter. Relative tumor proliferation rate was calculated using the formula: *R*_v _= (V_drug_/V_saline_) × 100%. Where V_drug_ is the relative glioma volume after treatment with drug, and V_saline_ is the relative glioma volume after treatment with physiological saline. The remaining 6 mice in each group were used for monitoring survival. Survival time was calculated from day 0 (tumor inoculation) to the day of death. Kaplan–Meier survival curves were plotted for each group. Meanwhile, the body weight of each mouse was measured every day.

#### Statistical analysis

The data are presented as the means ± standard deviation (SD). The difference between any two groups was determined via ANOVA. *p* < .05 was considered to be statistically significant.

## Results and discussion

### Synthesis of functional conjugates

**Figure 1.**
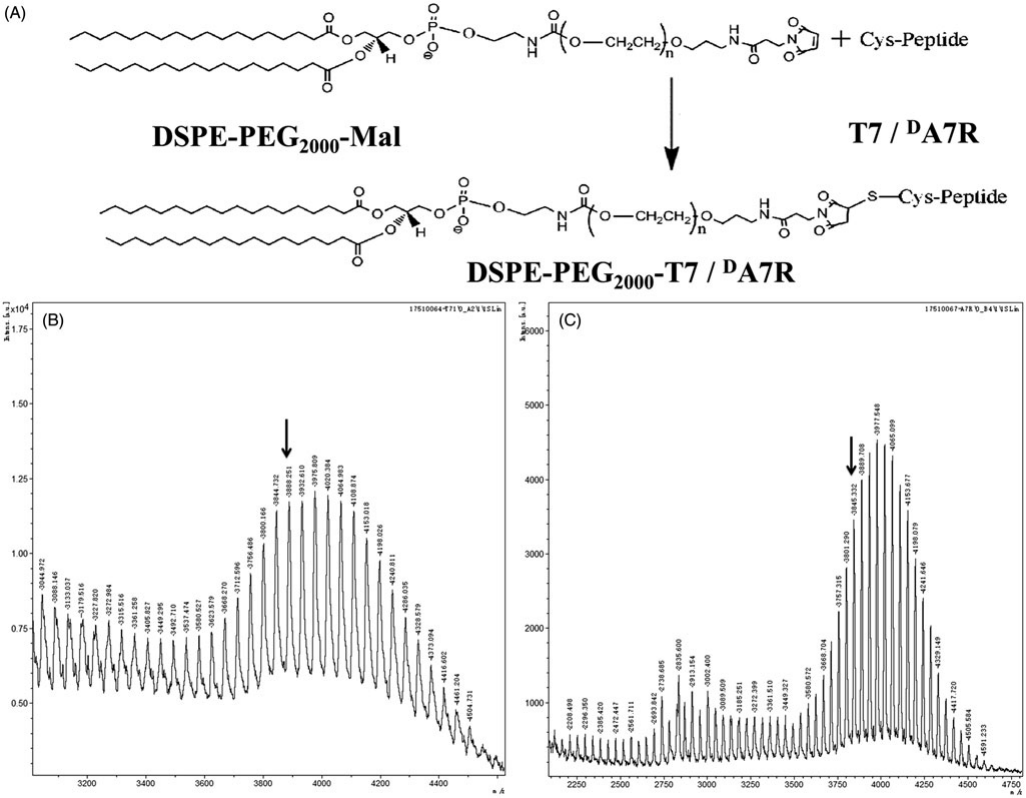
Principle of the synthesis of DSPE-PEG2000-T7/dA7R (A). MALDI-TOF mass spectra of DSPE-PEG2000-T7 (B) and DSPE-PEG2000-dA7R (C).

The T7/^D^A7R-LS was developed by the modification of two synthesized functional materials, DSPE-PEG_2000_-T7 and DSPE-PEG_2000_-^D^A7R. These functional materials were synthesized as shown in Figure 1. The T7 and ^D^A7R were terminated with cysteine to introduce free sulfhydryl (–SH), and these materials were conjugated to DSPE-PEG_2000_-Mal via the sulfhydryl-maleimide reaction, which enabled T7 or ^D^A7R to be conjugated at a specific site (–SH) (Figure 1(A)). The MALDI-TOF MS results confirmed the successful formation of DSPE-PEG_2000_-T7 and DSPE-PEG_2000_-^D^A7R, with the observed mass-charge ratios of approximately 3888 (Figure 1(B), marked by an arrow) and 3845 (Figure 1(C), marked by arrow), which was equal to the theoretical mass-charge ratios of 3888 and 3835, respectively. The final product was then used for preparing targeted liposomes in experiments.

### Characterization of liposomes

**Figure 2.**
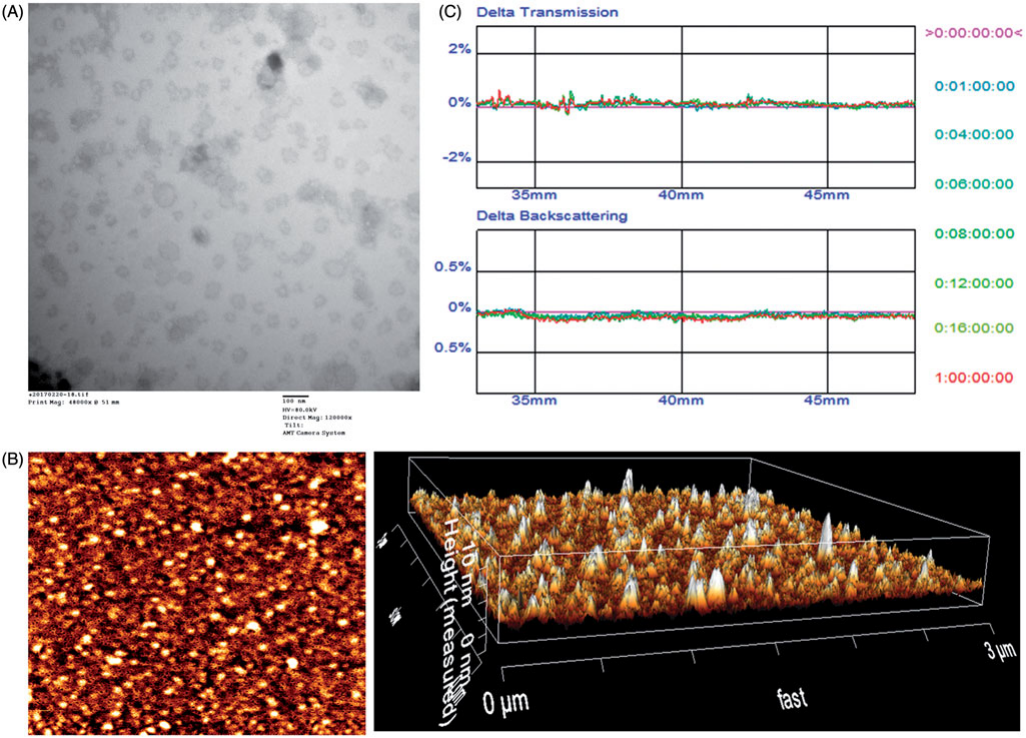
Physicochemical characterization of T7/dA7R-LS containing DOX and VCR. Morphological appearance of T7/dA7R-LS based on TEM (A) and AFM (B). Stability of T7/dA7R-LS in the presence of 10% FBS. The transmission and backscattering profiles were measured at each time point using a Turbiscan Lab^®^ Expert analyzer (C).

The physico-chemical properties of the four distinct liposome formulations are summarized in Table 1. The VCR and DOX EE of all liposomes were more than 85%, and the modifications of PSP or CPP on the surfaces of the liposomes did not affect the ultimate encapsulation efficiency. For drug delivery system (DDS), nanoparticle size would be a precondition and a crucial factor which decided the fate of DDS both *in vivo* and *in vitro*. After EE study, the particle size of DOX-loaded APO was further analyzed by laser particle analyzer. As shown in Table 1, the sizes of the N-DV-LS, T7-DV-LS, ^D^A7R-DV-LS, and T7/^D^A7R-DV-LS were between approximately 93.26 ± 0.10 and 95.87 ± 0.15 nm. We concluded that the sizes of the N-LS, T7-LS, ^D^A7R-LS, and T7/^D^A7R-LS were not significantly affected by the T7 or ^D^A7R modification. As shown in Figure 2(A,B), TEM and AFM images of T7/^D^A7R-LS demonstrated that the particle sizes were similar to those determined using a laser particle analyzer (90–100 nm).

**Table 1.** Characteristics of the liposomes.

Sample ID	Liposomes components (mol ratio of total lipid)	Diameter (nm)	Polydispersity index	DOX EE (%)	VCR EE (%)
N-LS	HSPC/Chol/DSPE-PEG_2000_ (50: 43: 7)	94.58 ± 0.11	0.08 ± 0.01	87.6 ± 2.0	88.3 ± 1.9
T7-LS	HSPC/Chol/DSPE-PEG_2000_/DSPE-PEG_2000_-T7 (50:43:4:3)	93.26 ± 0.10	0.09 ± 0.02	88.9 ± 2.1	87.9 ± 2.4
^d^A7R-LS	HSPC/Chol/DSPE-PEG_2000_/DSPE-PEG_2000_-^d^A7R (50:43:4:3)	95.30 ± 0.12	0.08 ± 0.01	87.2 ± 2.1	85.8 ± 1.9
T7/^d^A7R-LS	HSPC/Chol/DSPE-PEG_2000_/DSPE-PEG_2000_-^d^A7R/DSPE-PEG_2000_-T7 (50:43:1:3:3)	95.87 ± 0.15	0.10 ± 0.01	88.4 ± 1.8	86.4 ± 2.0

The data are expressed as the mean ± SD for three different preparations (*n* = 3).

The T7/^D^A7R-LS containing DOX and VCR stability against physiological conditions is a prerequisite for further application *in vivo*, and thus, 10% FBS in PBS was employed to mimic the *in vivo* situation. The *in vitro* stability of the T7/^D^A7R-DV-LS containing DOX and VCR in the 10% FBS was evaluated using Turbiscan Lab^®^ Expert. According to this judgment (Celia et al., 2009), the transmission or back-scattering profiles (less than 0.5%) obtained (Figure 2(C)) indicating there was no apparent aggregation or sedimentation occurred of T7/^D^A7R-LS containing DOX and VCR in the culture medium during 24 h. The instability phenomenon happened at 36 h (Figure S2).

### Optimization of peptide density of liposomes

**Figure 3.**
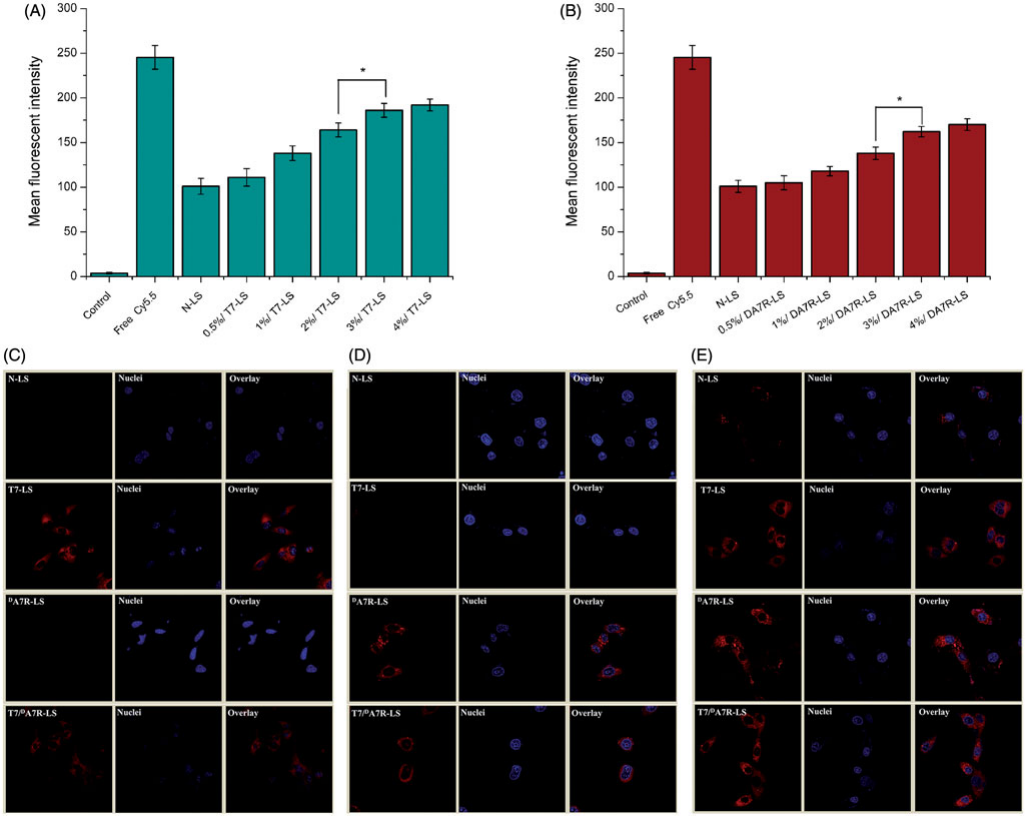
Cellular uptake of Cy5.5-loaded liposomes with different densities of T7 (A) and DA7R (B) in C6 cells after incubation for 2 h at 37 °C. The autofluorescence of the cells was applied as the control. Cellular uptake of different Cy5.5-loaded liposomes by bEND.3 cells (C), HUVECs (D), and C6 cells (E). The data are presented as the means ± SD (*n* = 3). *indicates *p* < .05.

As the density of T7 and ^D^A7R density in liposomes was a key factor that will influence the targeting efficiency of T7/^D^A7R-LS greatly, the cellular uptake of Cy5.5-loaded liposomes with modifications of different densities of peptides were evaluated in C6 cells to guide the formulation optimizing process. Glioma cells C6 were constantly chosen as the model of brain tumor cells because C6 glioma was very close to human multiform glioblastoma by morphology, characteristics of invasive growth, and spectrum of expressed proteins. In addition, C6 cells highly express both TfR and VEGFR 2 (Binetruy-Tournaire et al., 2000; Niu & Chen, 2010). Therefore, we chose C6 cells as the cell model of formulation optimization *in vitro*. As shown in Figure 3(A,B), when the peptide/lipid molar ratio was 0.5%, T7-LS, and ^D^A7R-LS both showed no significant increase of uptake compared with LS (*p* > .05). While, the cellular uptake of liposomes was significantly influenced by the increase of peptide/lipid molar ratio from 1 to 3%. With the further increase of the ratio to 4%, there was no remarkable difference in uptake compared with the liposomes with a 3% ratio. This was possibly caused by both the saturation phenomenon of TfR and VEGFR 2 on cells. Limited by the number of receptors and the recycling of endocytosis, receptor mediated endocytosis is a saturated pathway, which restricts the amount of the nanocarriers that are available for cellular uptake. Considering the above results, the molar ratio of 3% for both T7 and ^D^A7R was selected in next experiments.

### Binding of different liposomes with cells

To determine whether the affinity of liposomes to cells exhibits a difference after modification, various Cy5.5-loaded liposomal formulations were incubated with bEnd.3, HUVECs and C6 cells at 37 °C for 2 h, respectively. bEnd.3 cells, the main component of the BBB (Hu et al., 2009), was selected as the TfR-positive cell type used to investigate the effect of T7. HUVECs overexpressing VEGFR 2 were used as the models of tumor angiogenesis to confirm the neovasculature targeting ability of ^D^A7R (Zhu et al., 2011).

As shown in Figure 3(C), N-LS and ^D^A7R-LS could not efficiently recognize and bind with bEnd.3 cell, and thus, the uptake efficiency of N-LS and ^D^A7R-LS was not ideal based on the results of CLSM. The intracellular fluorescence of ^D^A7R-LS declined to a level similar to that of N-LS. On the contrary, both T7-LS and T7/^D^A7R-LS could significantly internalize into bEnd.3 cells in comparison to N-LS and ^D^A7R-LS, indicating that T7 functionalization on the liposomal surface could enhance brain targeting of liposomes. According to the design strategy, the dual-modified liposomes could efficiently inhibit glioma growth by crossing the BTB via the ^D^A7R motif. To verify this hypothesis, a VEGFR 2-positive HUVECs cell line was used to measure the cellular uptake of ^D^A7R functionalized liposomes, including ^D^A7R-LS and T7/^D^A7R-LS. As expected, ^D^A7R-LS and T7/^D^A7R-LS exhibited stronger intracellular fluorescence than N-LS and T7-LS in HUVESCs, which revealed the contribution of ^D^A7R in the ^D^A7R-LS or T7/^D^A7R-LS to cellular uptake (Figure 3(D)). Taken together, the synergistic effect of the two ligands on cellular uptake was clear. To evaluate the glioma-targeting efficiency, C6 cells were used to investigate the uptake of liposomes. As shown in Figure 3(E), both T7 functionalized liposomes (T7-LS and T7/^D^A7R-LS) and ^D^A7R functionalized liposomes (^D^A7R-LS and T7/^D^A7R-LS) displayed significant internalization into C6 cells, compared with those treated with N-LS. The results suggested that T7 and ^D^A7R functionalization on the surface of liposomes had significant influence on the tumor homing capacity of liposomes.

The uptake of various peptides-modified liposomes was further evaluated after pre-incubation with excess free T7 (1 mg/mL) and/or ^D^A7R (1 mg/mL) to saturate the cell surface receptors. As shown in Figure S3(A,B), the results suggest that the cellular uptake of various peptides-modified liposomes in the presence of excess free peptides in three cells was suppressed significantly, becoming almost equivalent to that of N-LS. These results confirmed the role of T7 and ^D^A7R peptide in the cellular uptake of liposomes and indicated that the enhancement of uptake result from the involvement of receptor-mediated endocytosis, namely by the interaction between the peptides and their receptors on tumor cells. Overall, these cellular uptake results strongly supported our hypothesis that the T7 and ^D^A7R can play a key role in the enhancement of cell recognition and uptake and the reduction of nonspecific cellular uptake.

### *In vitro* cytotoxicity of different liposomes

**Figure 4.**
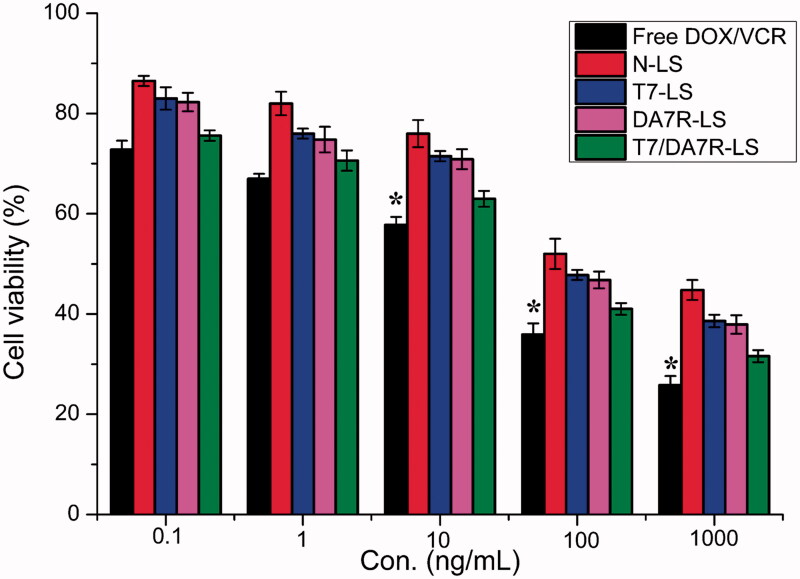
The cytotoxicity of free DOX + free VCR, and various liposomes containing DOX and VCR. The data are presented as the means ± SD (*n* = 3). *indicates *p* < .05.

MTT assay was conducted to evaluate the *in vitro* cytotoxicity of various liposomal formulations containing DOX and VCR in C6 cells. As shown in Figure 4, free DOX + free VCR could result in obvious antiproliferative effects to C6 cells in a concentration-dependent manner, thus proving the anticancer effect on such kind of brain tumors. In addition, the free DOX + free VCR group displayed the greatest cytotoxicity (IC_50_ values of 3.15 μg/mL) in C6 cells. It could be concluded from the results that free drug could be quickly transported into cells by passive diffusion with high concentration gradient under *in vitro* conditions. On the contrary, drug-loaded liposomes had undergone the drug release process after entering the intracellular region. So free DOX + free VCR exhibited stronger inhibitory effect to the proliferation of monolayer C6 cells compared with various liposomal formulations. Among these liposomes, the improved cellular uptake led to an anticipated enhanced anti-proliferation effect. This showed that the co-delivery of drugs (DOX + VCR) by the T7/^D^A7R-LS significantly increased the cytotoxicity, with an IC_50_ of 3.54 μg/mL, compared to 4.12 , 4.09, and 4.6 μg/mL for T7-LS, ^D^A7R-LS, and N-LS, respectively. The cytotoxicity studies demonstrated that the synergistic effect of T7 and ^D^A7R on the modified liposomes promoted anti-proliferative activities in C6 cells that overexpressed TfR and VEGFR 2.

### Penetration across the *in vitro* BBB and BTB models

BBB is a major physiologic barrier preventing drugs or drug delivery systems from getting into the brain-targeted region. Therefore, an *in vitro* co-culture model of bEnd.3/C6 cells was constructed to estimate the penetration efficiency of liposomes in mimicking conditions *in vivo*. After addition of the free DOX + free VCR, N-LS, ^D^A7R-LS, T7-LS, and T7/^D^A7R-LS, the survival (%) of C6 cells after crossing the bEnd.3 cells was 97.88 ± 2.53, 92.86 ± 3.33, 91.14 ± 1.74, 39.64 ± 2.94, and 40.05 ± 2.12%, respectively (Figure S4). The results indicated that both T7-LS and T7/^D^A7R-LS exhibited a significant inhibitory effect by transporting drug across the BBB and then targeting glioma cells. While, the free DOX + free VCR did not cross the BBB model at all. BTB was another characteristic pathological obstacle to the delivery of nanocarriers. To better imitate the BTB, an HUVECs/C6 cells co-culture model was set up to explore the targeting ability and transcytosis efficiency of various liposomes. As expected, ^D^A7R-LS and T7/^D^A7R-LS significantly increased the cytotoxicity of C6 cells after crossing the HUVECs, with a survival (%) of 41.88 ± 2.94% and 40.82 ± 1.87%, compared to 98.26 ± 0.93, 90.11 ± 1.02, and 92.47 ± 1.23% for free DOX + free VCR, N-LS, and T7-LS, respectively. This result suggested that liposomes with ^D^A7R modification possessed the targeting ability to the C6 cells after crossing the BTB. This result further supported the data of survival analysis in the BBB model. Although free DOX + free VCR could inhibit the growth of monolayer C6 cell more strongly than liposomes in the results of *in vitro* cytotoxicity assay (Figure 4), T7 or ^D^A7R functionalized liposomes could inhibit the growth of C6 cells after crossing bEnd.3 cells or HUVECs more strongly than free DOX + free VCR, which could be attributed to peptide functionalized liposomes targeting ability.

### Distribution of different liposomes in mice with intracranial glioma

**Figure 5.**
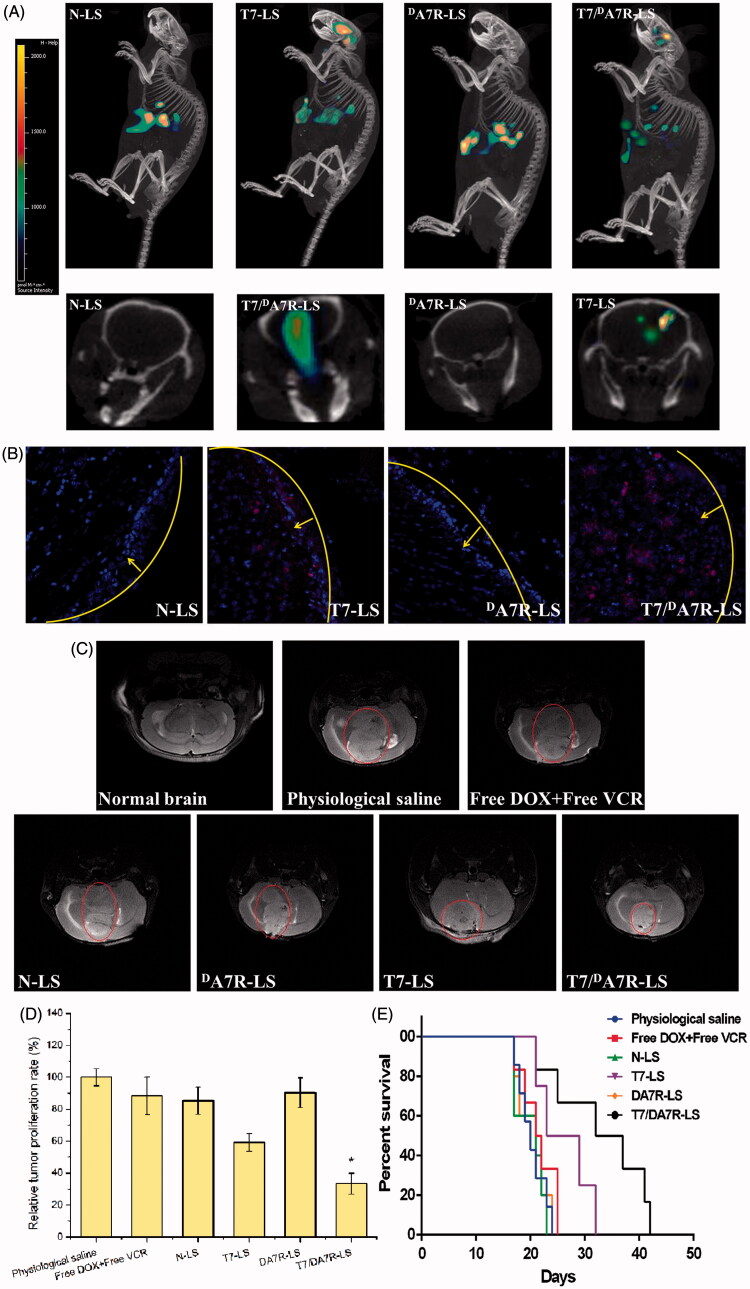
Biodistribution of Cy5.5 contained in various liposomes in mice bearing intracranial C6 glioma determined by an IVIS^®^ Spectrum-CT (A). Distribution of Cy5.5 in the brain of mice bearing intracranial C6 glioma determined by a CLSM (B). MRI of normal and pathological brains at 16 d after inoculation (C). Relative tumor proliferation rate of the brain glioma (D). Kaplan–Meier survival curves (E). The yellow line shows the margin of intracranial glioma and arrow indicates the glioma cells. The red represents Cy5.5 and the nuclei were stained by DAPI (blue). The data are presented as the means ± SD (*n* = 6). *indicates *p* < .05. Notes: Efficacy after treatment with various formulations with a dose of 1 mg/kg (DOX 0.8 mg/kg + VCR 0.2 mg/kg) at days 8, 10, 12, and 14 from inoculation.

The clinical therapeutic effect of glioma by drug treatment is very unsatisfying because of the existence of the multiple physiological barriers and non-targeted nature of drugs. The selective distribution of drug-formulated nanocarrier in tumors could potentially benefit the antitumor activity of chemotherapy *in vivo*. To verify this, in this study, the *in vivo* biodistribution of various Cy5.5-loaded liposomes was clearly visualized by monitoring the whole body fluorescence intensity in mice with intracranial C6 glioma. Based on whole body imaging (Figure 5(A)), the brain accumulation was much higher for the T7-LS and T7/^D^A7R-LS groups. While, there were no signals in the brain of animals treated with N-LS and ^D^A7R-LS. These initial data provided substantial evidence that T7 functionalized liposomes (T7-LS and T7/^D^A7R-LS) efficiently crossed the BBB and exhibited good brain targeting ability *in vivo*.

To further evaluate its *in vivo* glioma targeting capability, immunofluorescence assay was conducted after the treatment of various Cy5.5-loaded liposomes in mice bearing intracranial glioma. As shown in Figure 5(B), no fluorescence signals of N-LS and ^D^A7R-LS were observed in the glioma. In the T7-LS group, fluorescent intensity was uniformly distributed in the whole brain, suggestive of T7-LS across the BBB. T7/^D^A7R-LS showed a slightly higher accumulation than T7-LS in the brain but selective distribution in the glioma region, indicating the precise glioma targeting property of T7/^D^A7R-LS with the modification of both ligands. These results are consistent with the *in vivo* imaging results (Figure 5(A)) and indeed support our hypothesis that the T7/^D^A7R-LS could not only cross the BBB but also cross the BTB and selectively target the glioma cells. The results again emphasized the advantage of the dual-modified liposomes in brain targeting delivery.

Figure 5(A,B) showed almost no signal in the ^D^A7R-LS only group. It appears from this figure that the liposomes need to include T7 to target the brain. ^D^A7R-LS were effective *in vitro* (Figure 4), but they were unable to target glioma *in vivo*, this may be due to inadequate *in vivo* BBB penetrating activity of ^D^A7R-LS. The d-peptide ^D^A7R can efficiently across the BTB into glioma cells but they cannot effectively cross over the *in vivo* BBB.

### *In vivo* therapeutic efficacy

The *in vivo* antiglioma efficacy was investigated using the mice bearing intracranial C6 glioma. After treatment with the control formulations (T7-LS, ^D^A7R-LS, N-LS, free DOX + free VCR, and 0.9% saline), and T7/^D^A7R-LS, overall antiglioma efficacy was observed by MRI for monitoring cancer volume and was confirmed using survival curves. Consistent with the results of glioma distribution (Figure 5(A)), tumor inhibition analysis confirmed the significant brain glioma-targeting effect of T7/^D^A7R-LS in mice with intracranial C6 glioma. As shown in Figure 5(C), glioma diameter in the brain at day-16 was clearly reduced according to MRI after treatment with the T7/^D^A7R-LS as compared with those after treatment with control formulations, suggestive of T7/^D^A7R-LS across the multiple physiological barriers and targeting glioma cells. Relative tumor proliferation rate at day 16 (Figure 5(D)) was 100.00 ± 5.28% for physiological saline, 88.44 ± 11.65% for free DOX and free VCR, 85.31 ± 8.52% for N-LS, 59.31 ± 5.52% for T7-LS, 90.31 ± 9.46% for ^D^A7R-LS, and 33.31 ± 6.52% for T7/^D^A7R-LS. These results indicate that the therapeutic efficacy of the T7/^D^A7R-LS is significantly superior to that of other formulations in intracranial C6 glioma-bearing mice models. When the T7/^D^A7R-LS transported through BBB, part of T7 peptides may still remained in the liposomes, because as references (Kuang et al., 2013; Kang et al., 2015) reported that sole T7 peptide could navigate the delivery system across both BBB and BTB into the glioma cells. To enhance the BTB crossing and glioma cell targeting abilities of the delivery system, ^D^A7R was coupled to the carrier in this article. The above results confirmed this hypothesis.

The clinical therapeutic benefits are mainly determined based on the quality of life and prolonged survival time of cancer patients. In further investigation of the potential of T7/^D^A7R-LS in anti-glioma therapy i*n vivo*, the Kaplan–Meier survival curve of intracranial C6 glioma-bearing mice was used (Figure 5(E)). As expected, treatment with T7/^D^A7R-LS significantly prolonged the median survival time (34 d), which was 1.7, 1.6, and 1.3-fold higher than that of physiological saline, free DOX + free VCR, and T7-LS, respectively. This was mainly attributed to the target systemic delivery of T7/^D^A7R-LS, which was demonstrated by *in vivo* imaging (Figure 5(A,B)).

The goal for a drug delivery system is to achieve optimal therapeutic efficacy with acceptable safety profiles during *in vivo* applications. With respect to safety evaluation, the body weight variation of mice was monitored during the experimental period (Figure S6). More than 15% of weight loss was found in the free DOX + free VCR group at the end of experimental period. The weight loss of these free drug groups was likely due to the non-targeted characteristics and tumor cachexia. While, the much smaller of weight loss of mice in T7/^D^A7R-LS group than that of free DOX + free VCR group during the whole experimental period, indicated the dual-modified liposomes reducing unspecific cellular uptake through brain-targeted delivery.

As we known, transcytosis is a type of transcellular transport, in which various macromolecules are transported across the interior of a cell. In such pathway, macromolecules are encapsulated in vesicles on one side of a cell, then drawn across the cell, and released on the other side of the cell. Blood capillaries are a well-known site for transcytosis (Williams et al., 1984). Macromolecules as transferrin (Fishman et al., 1987) is transported through this way. As T7 peptide could bind to transferrin receptors, thus T7 modified liposomes could active the transcytosis pathway to transport drugs through the BBB. Thus, the dual modified liposomes in this article would across healthy BBB cells and target to the cancer cells.

## Conclusion

In this study, we successfully developed the dual-ligand liposomes modified with T7 and ^D^A7R. This system could penetrate BBB and BTB. Based on the glioma-targeted ability of T7/^D^A7R-LS, we have made full use of the synergistic effects of DOX and VCR in glioma suppression, and the restriction of their toxicity. Although preliminary, our study demonstrated a new avenue for treatment and experimental investigation of glioma and encourages further studies on the application of the dual-modified liposomes as an efficient delivery system of therapeutic agents in glioma chemotherapy. In a future study, we will continue to perform *in vitro* and *in vivo* evaluations, including the mechanism of targeted delivery, and further explore the application of T7/^D^A7R-LS in glioma-targeted delivery.
